# Insufficient exercise intensity for clinical benefit? Monitoring and quantification of a community-based Phase III cardiac rehabilitation programme: A United Kingdom perspective

**DOI:** 10.1371/journal.pone.0217654

**Published:** 2019-06-13

**Authors:** Alaa Khushhal, Simon Nichols, Sean Carroll, Grant Abt, Lee Ingle

**Affiliations:** 1 Department of Sport, Health & Exercise Science, University of Hull, Kingston-upon-Hull, United Kingdom; 2 Centre for Sport and Exercise Science, Sheffield Hallam University, Sheffield, United Kingdom; EHESP Paris, FRANCE

## Abstract

**Background:**

In recent years, criticism of the percentage range approach for individualised exercise prescription has intensified and we were concerned that sub-optimal exercise dose (especially intensity) may be in part responsible for the variability in the effectiveness of cardiac rehabilitation (CR) programmes in the United Kingdom (UK). The aim was to investigate the fidelity of a structured Phase III CR programme, by monitoring and quantifying exercise training intensity.

**Design:**

Observational study.

**Methods:**

The programme comprised 16 sessions over 8 weeks, where patients undertook an interval, circuit training approach within national guidelines for exercise prescription (40–70% heart rate reserve [HRR]). All patients wore an Apple Watch (Series 0 or 2, Watch OS2.0.1, Apple Inc., California, USA). We compared the mean % heart rate reserve (%HRR) achieved during the cardiovascular training component (%HRR-CV) of a circuit-based programme, with the %HRR during the active recovery phases (%HRR-AR) in a randomly selected cohort of patients attending standard CR. We then compared the mean %HRR-CV achieved with the minimal exercise intensity threshold during supervised exercise (40% HRR) recommended by national governing bodies.

**Results:**

Thirty cardiac patients (83% male; mean age [SD] 67 [10] years; BMI 28.3 [4.6] kg∙m^-2^) were recruited. We captured 332 individual training sessions. The mean %HRR-CV and %HRR-AR were 37 (10) %, and 31 (13) %, respectively. There was weak evidence to support the alternative hypothesis of a difference between the %HRR-CV and 40% HRR. There was very strong evidence to accept the alternative hypothesis that the mean %HRR-AR was lower than the mean %HRR-CV, median standardised effect size 1.1 (95%CI: 0.563 to 1.669), with a moderate to large effect.

**Conclusion:**

Mean exercise training intensity was below the lower limit of the minimal training intensity guidelines for a Phase III CR programme. These findings may be in part responsible for previous reports highlighting the significant variability in effectiveness of UK CR services and poor CRF improvements observed from several prior investigations.

## Introduction

Cardiovascular disease accounted for 15 million deaths worldwide in 2015 [1]. The cornerstone of secondary prevention strategies is comprehensive cardiac rehabilitation (CR), including optimal medical therapy and lifestyle interventions which have been endorsed within United Kingdom (UK) [2,3], European [4], and North American [5] guidelines. The literature on supervised, structured exercise training as a key component of comprehensive CR has been less than convincing. The largest RCT of structured CR within the UK showed no benefits to all-cause mortality. In 2013, Sandercock *et al* [6] quantified exercise training volumes and changes in cardiorespiratory fitness (CRF) in 950 cardiac patients undertaking Phase III CR across 4 centers in the UK. Patients completed 6 to 16 (median 8) supervised exercise sessions. CRF improvements showed an overall mean increase of only 0.52 METs; a third the mean estimate (1.55 METs) reported from the investigators earlier systematic review of the international CR literature [7]. In 2016, Almodhy et al [8] conducted a meta-analysis of UK CR studies in order to determine if programmes could promote meaningful changes in CRF. It was concluded that UK studies provided approximately one-third of the exercise "dose", and produced gains in CRF less than half the magnitude reported in the wider international studies.^8^ There are also challenges regarding the reporting of CR studies; Mitchell and colleagues [9], published a comprehensive systematic review and meta-analysis reporting a lack of consensus in the consistency of reporting of exercise interventions in CR studies. This highlights ongoing issues regarding lack of standardisation indicating the need for further high quality, robust CR trials.

The FITT principle describes the four components of exercise prescription: Frequency, Intensity, Time (duration), and Type of exercise which combine to create the exercise “dose”. Of these, exercise intensity is arguably the most critical component for improving CRF, and is the least standardised in clinical practice [10]. In the UK, CR programmes follow traditional approaches by prescribing exercise intensity based on a percentage range of heart rate reserve (%HRR; heart rate reserve being the differences between the maximal and resting heart rate, often with an adjustment if a patient is prescribed a medication which impacts chronotropic response, e.g. beta blockers). This method is recommended by a number of national associations including the American College of Sports Medicine [11], and in the UK, the Association of Chartered Physiotherapists in Cardiovascular Rehabilitation (ACPICR) [12], and the British Association of Cardiovascular Prevention and Rehabilitation (BACPR) [3].

Typically, structured Phase III CR is delivered in a community setting with the aim of achieving 20–60 min of moderate intensity continuous or interval-based exercise, 3–5 times per week, alongside resistance-based training [12]. In addition, most comprehensive CR programmes would include an educational component, dietary advice, and psychological support [13]. Most often in the UK, patients undertaking a Phase III programme would initially participate in group-based circuit training, following an interval training approach. Typically, patients would alternate between cardiovascular (CV) training interspersed with a period of active recovery (AR). The initial work: rest ratio would be dependent upon initial risk stratification and knowledge of existing individual baseline fitness levels. The goal would be to increase the dose of exercise by increasing the CV component and removing AR stations based on individual progress, with the ultimate aim that the patient is able to undertake continuous moderate intensity exercise. Importantly, for improvements in CRF, the CV component prescribed should be at an exercise intensity sufficiently high enough to induce physiological adaptation. Training intensity is based on an initial prescribed training zone utilising heart rate (HR) responses and/or ratings of perceived exertion (RPE). UK guidelines recommend an exercise intensity between 40–70% heart rate reserve (HRR) [3,12]. For AR stations, exercise HR and RPE levels should drop below prescribed exercise intensity levels to allow patients a brief period of respite [12].

In recent years, criticism of the percentage range approach for individualised exercise prescription has intensified [13,14] and we were concerned that sub-optimal exercise dose (especially intensity) may be, in part responsible for the variability in the effectiveness of UK CR programmes, which has been previously reported [15]. To our knowledge, no previous study has investigated the fidelity of a structured Phase III CR programme by monitoring and quantifying the exercise intensity achieved during an overall 8-week programme. We aimed to compare the mean percentage heart rate reserve (%HRR) achieved during the cardiovascular training component (%HRR-CV) of the programme, with the %HRR during the AR phases (%HRR-AR). Moreover, we compared the mean %HRR-CV achieved with the minimal exercise intensity threshold (40% HRR) recommended by national governing bodies [3,11].

## Materials and methods

The study was approved by the North West National Health Service (NHS) Research Ethics Committee and institutional ethics committee prior to commencement of the study. All patients provided written informed consent before participating in the trial. The following patients were eligible to participate in the study: patients following myocardial infarction (MI), whose event had presented in the preceding 3–6 weeks, percutaneous coronary intervention (PCI) and coronary artery bypass graft (CABG) within the past 6 weeks, stable angina, valvular or aortic root repair, patients with devices such as pacemaker or implantable cardioverter defibrillator, and stable heart failure. We excluded patients who had orthopaedic or neurological limitations, unstable angina, New York Heart Association class IV heart failure, uncontrolled hypertension and diabetes, symptomatic hypotension, uncontrolled tachy-arrhythmias, and febrile illness. Patients were referred from their hospital doctor or general practitioner to a Phase III CR programme based in the North Eastern region of England in 2017 and 2018.

The standard, community-based Phase III CR programme included two sessions per week for eight weeks (16 sessions in total). Each training session consisted of a 45 minute structured exercise based activities (12 training hours over eight weeks). Each training session consisted of a 15-minute warm up at the beginning of each session followed by a circuit training programme consisting of 9 exercise stations (5 CV; 4 AR). The CV stations included: treadmill, static bike, step ups, sit-to-stand, and rowing. The AR stations included biceps curls, wall press, lateral arm raises, and leg curl exercises. The session concluded with a 10-minute cool down period. All patients were advised not to consume caffeine at least three hours before the training sessions. In the UK, maximum heart rate is routinely estimated as there is little provision to conduct a maximal cardiopulmonary exercise test prior to routine CR. The following formula for calculating heart rate reserve (HRR) was used which is consistently advocated by national governing bodies [3, 11]:

Maximal heart rate was estimated using the following equation; HRR = 206 –(age x 0.7) [16,17] Resting heart rate was then deducted in order to calculate the HRR. A further deduction of 20–30 beats per min was made in patients who were prescribed beta-blockers, the decision to deduct 20 or 30 beats is often based on a patient’s initial risk stratification [11]. Heart rate training zones were calculated between 40–70% HRR in accordance with national guidelines [3,11]. In UK practice, patients often wear a heart rate monitor exercise classes which staff monitor periodically during CV exercise stations.

In our investigation, each patient wore an Apple Watch (Series 0 or 2, Watch OS2.0.1, Apple Inc., California, USA) during each training session over the 8-week intervention. The Apple Watch uses photoplethysmysmography (PPT) to measure heart rate. PPT is a non-invasive technique which uses a sensor to measure changes in blood flow [18]. We have recently shown that the Apple Watch heart rate sensor has very good validity during walking activities, and good validity during jogging activities. However, the validity of the device decreases as exercise intensities increase towards maximal levels [19]. During each training session, patients wore the watch on their left wrist with the exception of one patient who wore it on their right wrist due to an existing tattoo on their left side. Each Apple Watch was connected via Bluetooth to an iPhone 5s or iPhone 6 (Apple Inc., California, USA). We used the ‘Workout’ app to measure heart rate nominally at five second intervals. A bespoke iPhone app was written by one of the co-authors (GA) to extract the raw heart rate and sampling time data from the ‘Health’ database on the connected iPhone. The bespoke app was written using the Swift 2.1 language in XCode 7.2.1, utilising the methods supplied by the HealthKit framework (Apple Inc., California, USA).

### Statistical analysis

Mean and standard deviation (SD) are reported for normally distributed data. The mean %HRR for CV training and AR stations are reported separately. Data analysis was conducted in JASP (JASP Team, 2018) using Bayesian statistical methods. Below we describe the statistical analysis for two comparisons: (1) comparing the mean %HRR during CV exercise time (%HRR-CV) against the lower bound of the recommended training intensity zone (40% HRR), and (2) comparing the mean %HRR-CV against the mean %HRR for AR stations (%HRR-AR). Patients were excluded from our analysis if they failed to complete less than 12 of 16 training sessions (<75% adherence rate).

#### Comparing %HRR-CV and 40% HRR

We compared %HRR-CV and 40% HRR both in the form of a hypothesis test using Bayes factors and as a parameter estimation for the posterior distribution of the standardised effect size. Our null hypothesis test compared the observed data against a null hypothesis of no difference between the mean %HRR-CV and 40% HRR-CV, which is the lower bound of the recommended training intensity zone [3,11]. For this analysis we conducted a Bayesian one-sample t-test, using a two-sided alternative hypothesis because it is unknown if the mean %HRR-CV is above or below this lower bound. Given our uncertainty of the effect, we assigned a broad weakly informative prior using a zero-centred Cauchy distribution with scale r = 1/√2.

#### Comparing %HRR-CV and %HRR-AR

The same process was used to compare the mean %HRR-CV and mean %HRR-AR. We wanted to examine if the AR sections of the training sessions were in fact of lower intensity (and by what magnitude) compared to the CV section. For this analysis, we used a Bayesian paired-samples t-test with the same prior distributions. However, given that we were expecting the AR stations to be of lower intensity than CV sections, we used a one-sided alternative hypothesis.

To describe the strength of evidence against the null hypothesis (or for the alternative hypothesis) we used the classification scheme of Jeffreys [20]. A Bayes factor between one and three was considered as weak evidence; three and 10, moderate evidence, and above 10 is considered strong evidence. To describe the magnitude of the observed standardised effect size, we use the classification scheme of Cohen [21], with 0.2, 0.5 and 0.8 representing small, moderate, and large effects, respectively. Uncertainty in the parameter estimation is quantified using 95% credible intervals.

## Results

Thirty cardiac patients [83% male; age (SD) 67.0 (10.0) years; body mass index (SD) 28.3 (4.6) kg∙m^-2^] were recruited to the Phase III CR programme. Of these, 87% were prescribed beta-blockers; 53% statins; 40% ACE-inhibitors; and 68% aspirin. These medications remained unchanged throughout the training intervention. Patients were randomly selected from a mixed cardiovascular disease aetiology: 11 patients had received a coronary artery bypass graft, 7 percutaneous coronary intervention, and 2 patients had undergone a mitral valve replacement. Amongst the non-surgical patients, 4 patients were post-myocardial infarction, 3 patients had diagnosed coronary heart disease, and 3 were diagnosed with chronic heart failure. Of the 30 patients who initially enrolled, 4 exhibited paroxysmal atrial fibrillation.

Twenty patients (67% of the population) completed all 16 sessions (twice a week for eight weeks) of the Phase III CR programme; the remaining 10 patients dropped out at various stages of the programme due to a deterioration in their health, or were unable to attend due to personal reasons. Our analysis is based on 21 patients who had a programme adherence ≥75% (completed a minimum of 12 of 16 sessions). In total, we monitored 370 individual exercise training sessions, although data from 5 exercise sessions on selected patients were lost due to technical problems. Overall our analysis is based on 332 individual training sessions.

**Fig 1:**
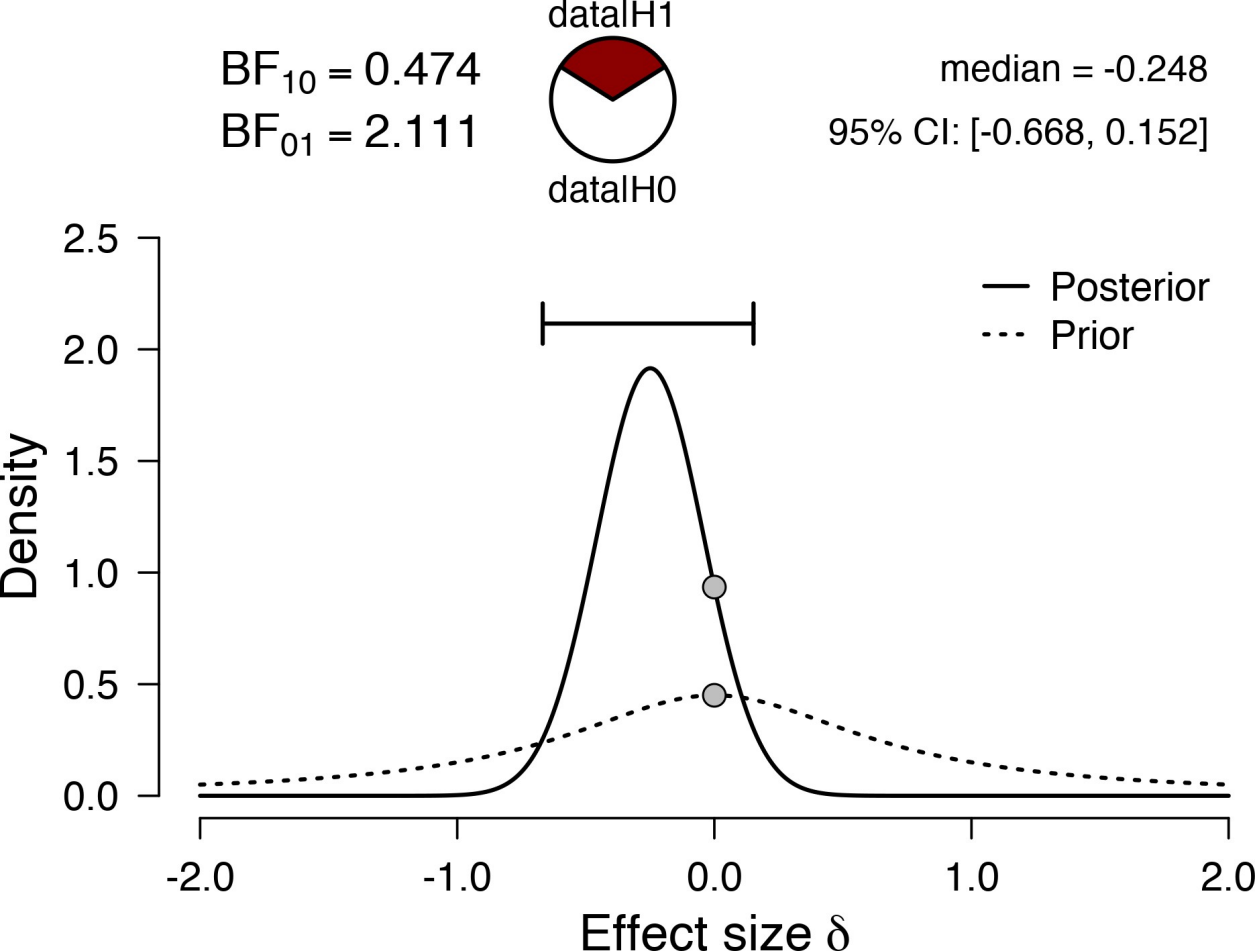
Prior and posterior effect size distributions, together with the effect size point estimate and associated 95% credible interval arising from a one-sample Bayesian t-test using a test value of 40. The value of 40 represents the lower bound of the recommended training intensity zone. The resulting Bayes_10_ factor provides weak evidence for the alternative hypothesis. BF = Bayes factor.

As displayed in Table 1, the mean (SD) %HRR-CV was 37 (10) %, with a 95% credible interval of 33 to 42%. The Bayesian one-sample t-test (Fig 1) resulted in a Bayes_10_ factor of 0.474, indicating weak evidence for the alternative hypothesis that the observed data is different from 40% HRR.

**Table 1:** Mean HR (beats per min) and %HRR for an 8-week Phase III circuit-based CR programme.

	Mean HR during CVE	Mean HR during AR	Mean %HRR during CVE	Mean %HRR during AR
Mean	90.6	87.0	37.1	31.5
Median	88.0	84.0	37.0	34.0
SD	12.3	13.2	10.1	12.6
Minimum	74.0	67.0	17.0	10.0
Maximum	117.0	113.0	62.0	53.0

HR = heart rate (bpm); CVE = cardiovascular exercise; AR = active recovery; %HRR = % heart rate reserve; SD = standard deviation

**Fig 2:**
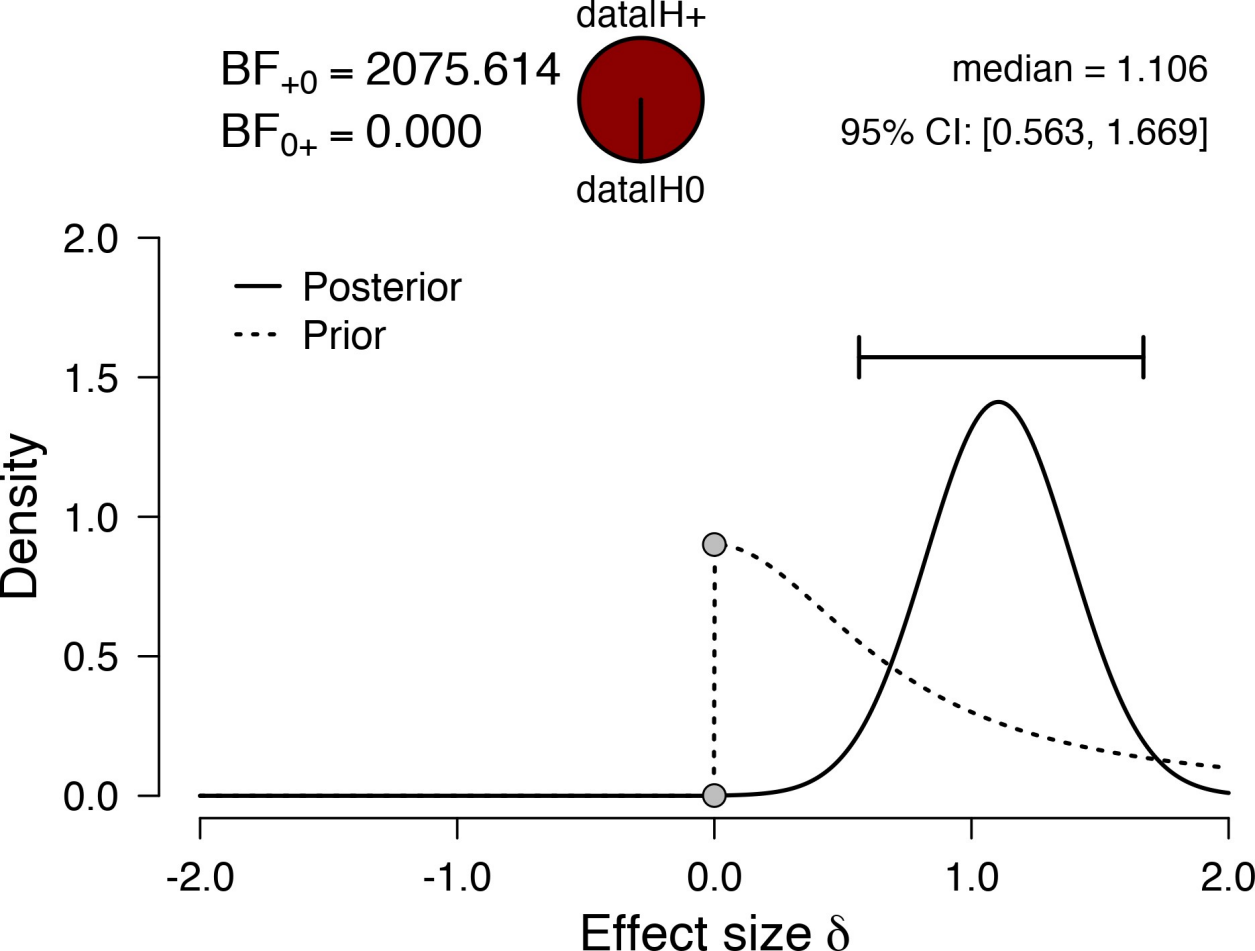
Prior and posterior effect size distributions, together with the effect size point estimate and associated 95% credible interval arising from a paired-sample Bayesian t-test examining the mean difference between %HRR-CV and %HRR-AR. The Bayes_10_ factor provides strong evidence for the alternative hypothesis that %HRR-AR is lower than %HRR-CV. The effect size of 1.1 suggests a large difference between the two means. BF = Bayes factor.

**Fig 3:**
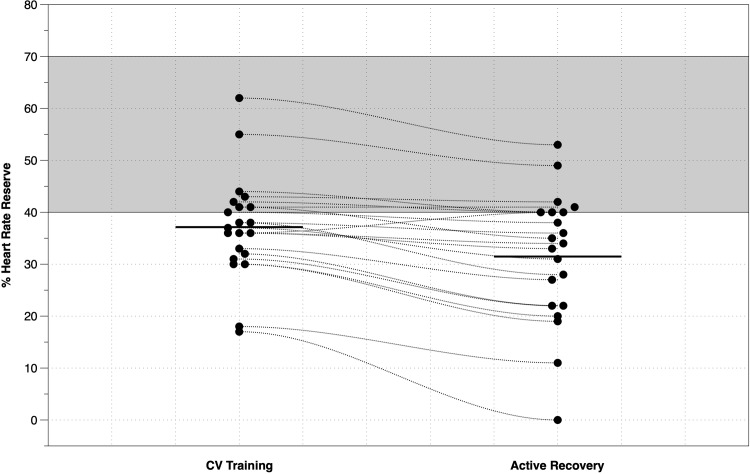
The means (horizontal bar) and individual mean distributions of CV and AR components of an 8-week Phase III CR interval training programme. The grey zone represents the recommended training intensity following national guidelines (3,12).

The mean (SD) %HRR-AR was 31 (13) %, which is approximately 6% lower than the mean %HRR-CV (Table 1). When comparing the mean %HRR-CV against the mean %HRR-AR (Fig 2), the Bayes_10_ factor of 2076 indicates very strong evidence in favour of the alternative hypothesis. The median standardised effect size of 1.1 (95%CI: 0.563 to 1.669) suggests a moderate to large effect. Fig 3 shows the overall mean and individual mean distributions of CV and AR components of an 8-week Phase III CR interval training programme. We noted that 67% of our patients had a mean HRR-CV% below 40% HRR, which is the lowest end of the exercise prescription guidelines advocated by national governing bodies.

## Discussion

To the best of our knowledge, our study is the first UK-based study to monitor and quantify exercise training intensity during a structured, community-based Phase III CR programme. Recent reports have highlighted that CR programmes in the UK may be less effective than international programmes 7,9] and may not attain a sufficient exercise intensity to promote physiological adaptations and corresponding improvements in CRF. Our findings provide further support for these assertions. The mean exercise intensity during the CV training component of the Phase III programme was only 37 (10) % HRR (mean HR achieved <91 [12] bpm) which is below, (but not statistically), the minimum national guidelines for training intensity [3,11]. Fig 3 highlights the large variability in mean training intensity received by patients on an individual basis, with 67% of our patients training at an intensity below the lowest threshold advocated by national governing bodies [3,11].

UK-based guidelines advocate a percentage range-based method for prescribing exercise intensity [3,11]. However, there are a number of limitations of this method; firstly, it fails to account for individual metabolic responses e.g. ventilatory and blood lactate responses to interval exercise [22], which is problematic given that exercise prescription is required to be personalised for patients with cardiac disease [11]. Examples of this lack of personalisation have been demonstrated in previous studies [23–25], which have shown considerable individual variation in blood lactate response to exercise when intensity is anchored to a relative percentage range. A heterogenous metabolic response to exercise training has been offered as a possible mechanism for the variability in effectiveness of exercise training programmes, thus resulting in positive responders and non-responders [26]. Wolpern et al [27] conducted a randomised controlled trial to compare the effectiveness of a threshold-based model (ventilatory threshold) versus a relative percent model (%HRR) for improving cardiorespiratory fitness in 36 males and females. They found that the threshold-based model elicited significantly (*P*<0.05) greater improvements in VO_2_max compared to a %HRR model following 12 weeks of training. However, an interesting finding from this study was that the threshold-based model attenuated the individual variation in VO_2_max training responses when compared to the %HRR group. The authors reported considerable heterogeneity in terms of responders (41.7%), and non-responders (58.3%) for eliciting changes in VO_2_max following training, which is consistent with other studies [22,28]. Wolpern et al [26] showed that in the threshold-based training group, 100% of participants demonstrated a positive improvement in VO_2_max following training. A mean improvement of 1.1 METs (full range: +0.65 to +1.63 METs) was reported among these young or middle aged previously sedentary groups. In contrast, only 41.7% of participants experienced a significant improvement in VO_2_max in the %HRR group.

In the UK, there are >300 registered CR programmes (Phase III and IV), which undoubtedly leads to issues in relation to consistency of service quality and delivery. In recent years, there have been some positive signs reported in relation to improved consistency of service provision. In 2015, the BACPR and National Association of Cardiac Rehabilitation (NACR) developed the National Certification programme for CR (NCP_CR) services, which sets out to improve delivery of CR, showcase good services, and seek to ensure the effectiveness of routine provision of CR programmes through achievement of a minimum level of service delivery across the UK [29,30].

Doherty and colleagues [15] recently conducted an audit to investigate how many UK CR programmes met minimal standards for delivery of the NCP_CR. The analysis used UK NACR data extracted and validated for the period 2013–2014 set against six NCP_CR measures recognised as important for the delivery of high-quality CR programmes. Data from 170 CR programmes revealed significant variability in terms of quality of service delivery; 30.6% were assessed as high performing, 45.9% as mid-level performing, 18.2% were classified as low performing, and 5.3% failed to meet any of the minimum criteria. These findings indicate that substantial variation, below the recommended minimum standards, exists throughout the UK. The six measures deemed important for high quality provision relate to the service being offered to all priority groups; ≥69% of patients with recorded assessments before starting a formal CR programme; ≥49% of patients with recorded assessment after completing a CR programme; median waiting time from referral to start of CR within 40 days (post-MI); median waiting time from referral to start of CR within 54 days (post-CABG); Median duration of CR programmes being 54 days for conventional delivery, or 42 days where the Heart Manual was used [15]. Currently, there are no criteria which relate to service effectiveness which, if incorporated, may assist with improving service outcomes at a national level.

A limitation of current UK practice is that maximum heart rate is estimated, and not directly measured. Subsequently, adjustment for beta-blockade is added, and then a resting heart rate (RHR) value is included in the calculation. Each of these steps incur error of estimation, reducing the accuracy of the training heart rate range which is prescribed to the patient [31]. Alternative and more valid equations for estimating heart rate maximum have been derived from specific cardiac cohorts which, crucially, have been adjusted for beta-blockade [32]. It may be that a following a similar approach should be a recommendation for UK CR services. The timing of the RHR assessment is also important: cardiovascular physiology appears to follow a daily biorhythm; heart rate, blood pressure, and cardiac contractility all peak in the wake hours and reach a nadir during sleep [33]. The suppressive effect of propranolol, for example, on the rise in HR during exercise is significantly greater if the drug is taken in the morning versus at night [34]. Therefore, to ensure minimal variability in the daily RHR, CR staff should be mindful that offering classes at regular times of the day will help minimise biorhythm disturbance. Secondly, checking patients timing and compliance of their beta-blocker medication will also assist with this goal. It is also possible that over time, cardiorespiratory may improve, potentially lowering the RHR. Furthermore, medication changes may also be responsible for an altered RHR. Therefore, CR staff should ensure that RHR is checked and recorded prior to the start of each training session in order to ensure a more precise estimation of the individualised HR training zone. Finally, the implementation of these processes are fundamental to effective practice, hence the requirement for ongoing professional development and education to ensure that CR service quality and standards are improved.

A limitation of our study is that our detailed evaluation of training intensity from a Phase III community-based CR programme is based on findings from a single NHS centre and may not be representative of exercise training intensities or prescription methods undertaken in other centres. We have already acknowledged the high levels of inconsistent and variable quality of CR service provision in the UK. It is highly probable that many of the >300 UK CR programmes would be far more effective with patient outcomes being significantly greater than in the CR service we observed. Conversely, however, it is also possible that some other CR programmes may be inferior in terms of patient outcomes, therefore, the key is trying to improve the consistency of CR service provision and quality across the UK.

Our study did not directly measure peak HR, rather it was estimated using a predictive equation, including the adjustment for beta-blockade. Therefore, we cannot say how accurately our training zone calculations were (compared to directly measured findings), however, we have systematically followed the process for estimating HR training zones as recommended by the BACPR and ACPICR in the UK. Consequently, we are confident that our study is pragmatic, and provides real-world application.

We utilised two models of Apple watch in our study. It is possible that this technical issue may have introduced some degree of systematic error to our findings as we are not aware of the differences in technical specification between the models.

In conclusion, within a heterogeneous cohort of patients with cardiovascular disease attending routine Phase III CR, mean exercise training intensity was below, but not significantly, the minimal exercise training intensity threshold (40% HRR) recommended within national guidelines in the UK. The generalisability of these findings requires further investigation. However, they may be in part responsible for previous reports highlighting the significant variability in effectiveness of UK CR services, and the poor improvements in CRF documented in patients undertaking CR in the UK compared to international standards.

## Supporting information

S1 File Data file. (CSV)
